# Zisheng Shenqi Decoction Ameliorates Monosodium Urate-Mediated Gouty Arthritis in Rats via Promotion of Autophagy through the AMPK/mTOR Signaling Pathway

**DOI:** 10.1155/2021/6918026

**Published:** 2021-01-06

**Authors:** Jieru Han, Guangyu Shi, Wenhao Li, Shuhui Wang, Jixiang Bai, Xutao Sun, Ying Xie, Fangyu Sui, Fei Chen, Deyou Jiang

**Affiliations:** ^1^Department of Synopsis of the Golden Chamber, School of Basic Medical Sciences, Heilongjiang University of Chinese Medicine, Harbin 150040, China; ^2^Department of Ultrasound Medicine, First Affiliated Hospital, Heilongjiang University of Chinese Medicine, Harbin 150040, China; ^3^Department of Integrated Chinese and Western Medicine, Department of Geratology, Hongqi Hospital Affiliated to Mudanjiang Medical University, Mudanjiang 157011, China; ^4^Department of Urology, Hongqi Hospital Affiliated to Mudanjiang Medical University, Mudanjiang 157011, China; ^5^Department of Chinese Herbs, School of Basic Medical Sciences, Heilongjiang University of Chinese Medicine, Harbin 150040, China

## Abstract

Gouty arthritis (GA) is an inflammatory disease owing to the accumulation of monosodium urate (MSU) in joints, leading to redness and burning pain. In this study, the effect of Zisheng Shenqi Decoction (ZSD) on a rat model of MSU-induced GA was investigated. ZSD obviously diminished the right paw thickness, the degree of the swelling of the paw, and the infiltration of the inflammatory cell, as well as cartilage erosion, and widened the joint space in MSU-treated rats. Besides, MSU remarkably elevated the release of tumor necrosis factor-*α* (TNF-*α*), interleukin-1*β* (IL-1*β*), IL-6, and IL-18; however, ZSD treatment dose dependently lowered these levels and resulted in a significant decrease in articular elastase activity. Also, ZSD administration increased the activities of superoxide dismutase (SOD), glutathione peroxidase (GSH-Px), and catalase (CAT) but declined malondialdehyde (MDA) and nitrogen monoxide (NO) contents. Importantly, western blotting analysis revealed that NOD-like receptor protein 3 (NLRP3), cleaved caspase-1, IL-1*β*, nuclear factor-E2-related factor 2 (Nrf2) in the cytoplasm, phosphorylated mammalian target of rapamyclin (p-mTOR), and p62 expressions were downregulated, whereas the levels of nuclear Nrf2, phosphorylated AMP-activated protein kinase (p-AMPK), Beclin-1, and LC3II/I were upregulated by ZSD. Immunofluorescence assay indicated that ZSD evidently promoted nuclear translocation of LC3. Taken together, ZSD inhibited inflammation and oxidative stress and facilitated autophagy through the activation of the AMPK pathway and suppression of the mTOR signaling pathway, demonstrating its potential for preventing and curing GA.

## 1. Introduction

Excessive alcohol intake, obesity, type 2 diabetes mellitus, hypertension, metabolic syndrome, chronic kidney disease, and the use of medications are capable of increasing the risk of gout [1]. Gout is a metabolic disease involved in joint pain, fatigue, and high fever, second only to diabetes in China [2, 3]. The previous report indicated that the gout incidence was 1.14% in the coastal cities of Shandong province [4]. Gout prevalence was closely related to age, especially in men more than 40 years old, concomitant with metabolic syndrome [5]. Gouty arthritis (GA) is an acute inflammatory response caused by formation and precipitation of monosodium urate (MSU) crystals in the articular and surrounding joints with high disability rate, which is the most common first clinical manifestation in gout [4]. Varieties of proinflammatory indicators such as tumor necrosis factor-*α* (TNF-*α*), interleukin-1*β* (IL-1*β*), IL-6, and IL-18 release are exacerbated through the deposition of MSU crystals, thereby resulting in intense pain and swelling, as well as articular inflammation [5]. Moreover, mast cells, monocytes/macrophages, and neutrophils take part in gouty inflammatory response. Among them, mast cells are associated with the early phase of MSU-induced inflammation [6]. Macrophages have a crucial role in the recognition of MSU [7]. Neutrophil influx in the synovium is the most important character of GA. Accumulation of neutrophils and synovial cells induces lysis of the membrane, which in turn generates reactive oxygen species (ROS) and facilitates lysosome release [8]. Until now, although colchicine, corticosteroids, and nonsteroidal anti-inflammatory drugs (NSAIDs) have been widely utilized for the treatment of GA, they still have some side effects containing renal toxicity, gastritis, and gastrointestinal bleeding [8, 9]. Therefore, it is vital to seek for novel and curative drugs to prevent and treat GA.

Zisheng Shenqi Decoction (ZSD) has been developed from Liuwei Dihuang (LWDH), a classic traditional Chinese herbal formula that includes six common herbs: *Radix Rehmanniae* (Dihuang; prepared root of *Rehmannia glutinosa*), *Rhizoma dioscoreae* (Shanyao; rhizome of *Dioscorea opposita*), *Fructus corni* (Shanzhuyu; fruit of *Cornus officinalis*), *Cortex moutan radicis* (Mudanpi; root bark of *Paeonia suffruticosa*), *Rhizoma Alismatis* (Zexie; rhizome of *Alisma plantago-aquatica*), and *Poria* (Fuling; sclerotia of *Poria cocos*) [10]. LWDH has been widely used for the treatment of kidney yin deficiency, type 2 diabetes mellitus, osteoporosis, and the neuroendocrine immunomodulation network [10, 11]. In addition, ZSD mainly consists of five medicinal herbs such as *Amomum villosum* (Fructus Amomi, Sha Ren), *Achyranthes bidentata* (Radix Cyathulae, Huai Niu Xi), *Semen Plantaginis* (plantain seed, Che Qian Zi), *Rhizoma Smilacis Glabrae* (Glabrous Greenbrier Rhizome, Tu Fu Ling), and *Dioscorea septemloba* (Sevenlobed Yam Rhizome, Bi Xie). ZSD was found to improve the kidney, remove dampness, contribute to blood circulation, and mitigate pain [12]. It was discovered that ZSD could effectively attenuate MSU crystal-mediated GA in rats via lowered production of IL-6, TNF-*α*, IL-1*β*, and NOD-like receptor proteins (NLRP1 and NLRP6) and elevated superoxide dismutase (SOD) and glutathione peroxidase (GSH-Px) activities [12]. However, the underlying mechanism that ZSD relieved MSU-induced GA has not been fully illuminated.

In the present study, we demonstrated that ZSD could activate the AMP-activated protein kinase (AMPK) signaling pathway and inhibit the mammalian target of the rapamyclin (mTOR) pathway to promote autophagy in a rat model of GA. Besides, ZSD reduced inflammatory response and inhibited oxidative stress (OS) in MSU-treated rats.

## 2. Materials and Methods

### 2.1. Animals

Six-week Wistar rats (230 ± 10 g) were provided with ad libitum access to food and water. They were acclimatized in a controlled room (22 ± 1°C, 12 h dark-light cycle, and 45–55% humidity). All animal studies and protocols were approved by the Laboratory Animal Use and Management Committee of Heilongjiang University of Chinese Medicine.

### 2.2. The Rat Model of MSU-Induced GA and ZSD Treatment

The preparation of MSU crystals was according to the previous method [6]. ZSD preparation and dose selection referenced the previous article [12]. The rats were randomly assigned into five groups (*n* = 6). First, ZSD (10 or 20 or 40 mg/kg/day; identified as MSU + ZSD-L, MSU + ZSD-M, and MSU + ZSD-H, respectively) was infused to the stomach of rats for 7 consecutive days. One hour after the 7th day of administration, rats were anesthetized with 2.5% isoflurane, and then MSU (10 mg/mL) was injected into the right ankle joint of rats. Next, the thickness of the right paw in rats was measured by using a vernier caliper for 0, 24, 48, and 72 h after MSU injection, respectively. Then, after the injection of MSU for 72 h, the swelling of the right paw in each group was observed and photographed. Finally, rats were anesthetized and sacrificed. Blood was sampled from the caudal vein of rats, and serum was separated and stored at −80°C until biochemical detection. The ankle synovial tissues were fixed and frozen used for subsequent experiments.

### 2.3. Histological Analysis

Five *μ*m-thick sections from paraffin-embedded samples were deparaffinized with xylene, dehydrated using a graded ethanol series, and incubated with hematoxylin for 5 min. The sections were counterstained with eosin dye for 3 min. All sections were visualized under the microscope (BX53, Olympus, Japan). Histological changes in the joint space, inflammatory cell infiltration, and cartilage erosion of rat ankles were scored in accordance with the previous research [13]. The histological scores were graded as 0–3 points: 0 = normal, 1 = mild mononuclear cell infiltration, 2 = moderate inflammatory cell infiltration and cartilage destruction, and 3 = massive pannus formation with extension of inflammatory cell invasion into the synovium and joint space.

### 2.4. Detection of Proinflammatory Cytokines and Articular Elastase

The concentration of TNF-*α* (SEA133Ra), IL-1*β* (SEA563Ra), IL-6 (SEA079Ra), IL-18 (SEA064Ra), and articular elastase (SEA181Ra) in serum of treated rats was measured with a microplate reader (ELX-800, BIOTEK, USA) by enzyme-linked immunosorbent assay (ELISA) kits (Uscn Kit Inc., Wuhan, China) according to the manufacturer's instructions.

### 2.5. Measurement of Factors Involved with the Antioxidant System and Oxidative Stress (OS)

The activities of GSH-Px (A005), SOD (A001-1), and CAT (A007) and contents of MDA (A003-1) and NO (A013-2) in serum of treated rats were detected via corresponding kits (Nanjing Jiancheng Bioengineering Institute, Nanjing, China) in accordance with the manufacturer's recommendations. In brief, the absorbance was measured at a wavelength of 412 nm (GSH-Px) or 550 nm (NO) by a microplate reader (M200PRO, TECAN, Switzerland); the absorbance was assessed at a wavelength of 550 nm (SOD), 532 nm (MDA), or 405 nm (CAT) via a UV-visible spectrophotometer (UV752N, Shanghai Yoke, China).

### 2.6. Quantitative Real-Time Polymerase Chain Reaction (qRT-PCR)

Total RNA was isolated from ankle joint tissue utilizing the TRIpure reagent (RP1001, BioTeke, China) following the manufacturer's instructions. The total RNA was reversely transcribed to cDNA, and subsequently, qRT-PCR was performed by ExicyclerTM 96 Real-Time PCR System (BIONEER, Korea) with SYBR Green (SY1020, Solarbio Science and Technology, China). Reaction conditions were as follows: step 1: 94°C for 5 min, step 2: 94°C for 10 s, step 3: 60°C for 20 s, and step 4: 72°C for 30 s and 40 cycles of 72°C for 2.5 min, 40°C for 1.5 min, melting 60°C to 94°C (1°C/s), and 25°C for 1-2 min. *β*-Actin was used as the internal control. The PCR results were quantified via the 2^−ΔΔCt^ method. The primer sequences used in this study were purchased from Genscript Biotechnology Co., Ltd (Nanjing, China) and are listed in Table 1.

### 2.7. Western Blotting

Protein was extracted from treated tissues by the whole protein extraction kit (WLA019, Wanleibio, Shenyang, China) and the nuclear protein and plasma protein kit (WLA020, Wanleibio) according to the manufacturer's instructions, respectively. In brief, ankle joint tissues were lysed in lysis containing 1% phenylmethylsulfonyl fluoride on ice for 5 min. After centrifugation (4°C, 12000 rpm, 10 min), the supernatant was collected as the total protein. The concentration of the protein was determined by the BCA kit (WLA004, Wanleibio). Proteins were electrophoresed on 5%, 8%, 10%, and 15% SDS-PAGE and then electrotransferred to PVDF membranes (IPVH00010, Millipore, USA). The membranes were blocked with 5% skimmed milk in TBST for 1 h and incubated with primary antibodies overnight at 4°C, respectively. After being washed with TBST, the membranes were incubated with the secondary antibody (37°C, 45 min). The blots were detected using the ECL kit (WLA003, Wanleibio) and quantified with the Gel-Pro-Analyzer system (WD-9413B, Liuyi, China). *β*-Actin and histone H3 were utilized as internal references, respectively. The antibodies used in this study were diluted with 5% skimmed milk, and the information is listed in Table 2.

### 2.8. Immunofluorescence Staining

Paraffin-embedded tissue sections of 5 *μ*m thickness were placed on slides, deparaffinized with xylene, and rehydrated with 95%, 85%, and 75% alcohol in sequence. The sections were placed in citrate buffer, repaired for 10 min, and blocked with goat serum (SL038, Solarbio Science and Technology) for 10 min at room temperature. The sections were then incubated with the Nrf2 rabbit antibody (16396-1-AP, dilution 1 : 50 in PBS, Proteintech Group Inc., China) or LC3 mouse antibody (sc-376404, dilution 1 : 50 in PBS, Santa Cruz, USA) overnight at 4°C. After washing with PBS, the slides were incubated with goat anti-rabbit IgG (A0516, dilution 1 : 200 in PBS, Beyotime Biotechnology, China) or goat anti-mouse IgG (A0521, 1 : 200, Beyotime Biotechnology) in the dark for 1 h. DAPI (C1002, Beyotime Biotechnology) was added to each slide for localization of nucleus, and the images were observed by using a fluorescent microscope.

### 2.9. Statistical Analysis

The results were presented as means ± SD. Differences in homogeneity of variances were calculated with Levene's test, and all statistical tests were analyzed by one-way or two-way analysis of variance (ANOVA) using Tukey's multiple comparison test with GraphPad Prism version 8.0. Scoring was analyzed with Dunnett's multiple comparison test. *P* < 0.05 was considered as significant difference.

## 3. Results

### 3.1. ZSD Attenuated MSU-Induced GA

**Figure 1:**
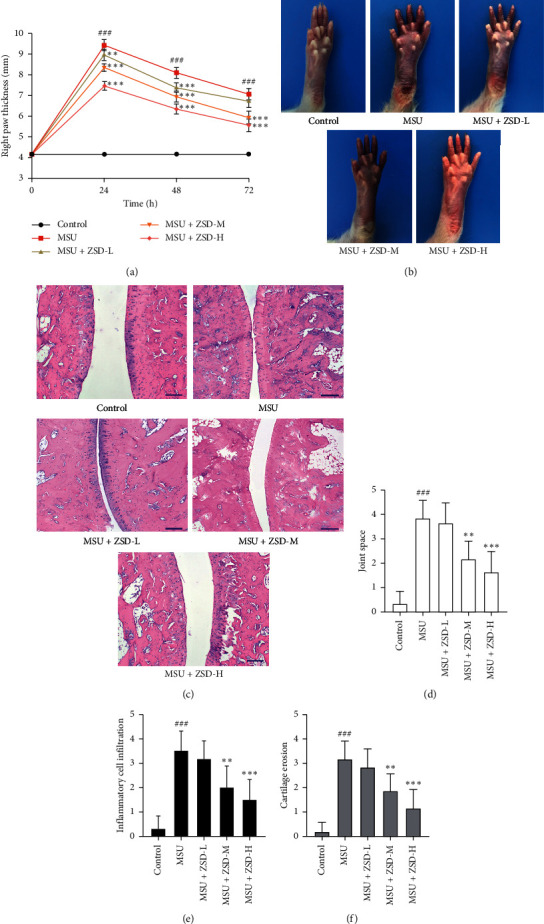
ZSD attenuated MSU-induced GA in rats. (a) Thickness of the right paw in each group was measured for 0, 24, 48, and 72 h after MSU injection, respectively. (b) After the injection of MSU for 72 h, the swelling of the right paw in each group was observed and photographed. Then, rats were anesthetized again and sacrificed. (c) The pathological changes in the ankle joint were detected by H&E staining. The scale bar = 200 *μ*m. ((d)–(f)) Histopathological changes in the joint space, inflammatory cell infiltration, and cartilage erosion were scored. Results were expressed as means ± SD (*n* = 6). ^###^*P* < 0.001 compared with the control group; ^∗∗^*P* < 0.01 and ^∗∗∗^*P* < 0.001 compared with the MSU group. ZSD: Zisheng Shenqi Decoction; MSU: monosodium urate; GA: gouty arthritis; H&E: hematoxylin and eosin.

To evaluate the extent of swelling, the thickness of the right paw was detected in control and treated rats. As shown in Figure 1(a), MSU injection strongly increased the right paw thickness at 24, 48, and 72 h. However, ZSD administration significantly decreased the thickness in a dose-dependent manner. Consistently, the MSU-mediated rats developed the swelling of the right paw, whereas pretreatment with ZSD obviously attenuated this process (Figure 1(b)). Furthermore, ankle joints were stained with H&E and visualized via using a microscope (Figure 1(c)). It was found that administration of MSU induced GA, while ZSD treatment widened the joint space and reduced the infiltration of the inflammatory cell and cartilage erosion. The scores for joint space, inflammatory cell infiltration, and cartilage erosion were also remarkably lowered by ZSD in the ankle joint of MSU-treated rats (Figures 1(d)–1(f)). These results suggested that ZSD inhibited MSU-induced GA.

### 3.2. ZSD Inhibited MSU-Induced Inflammation

**Figure 2:**
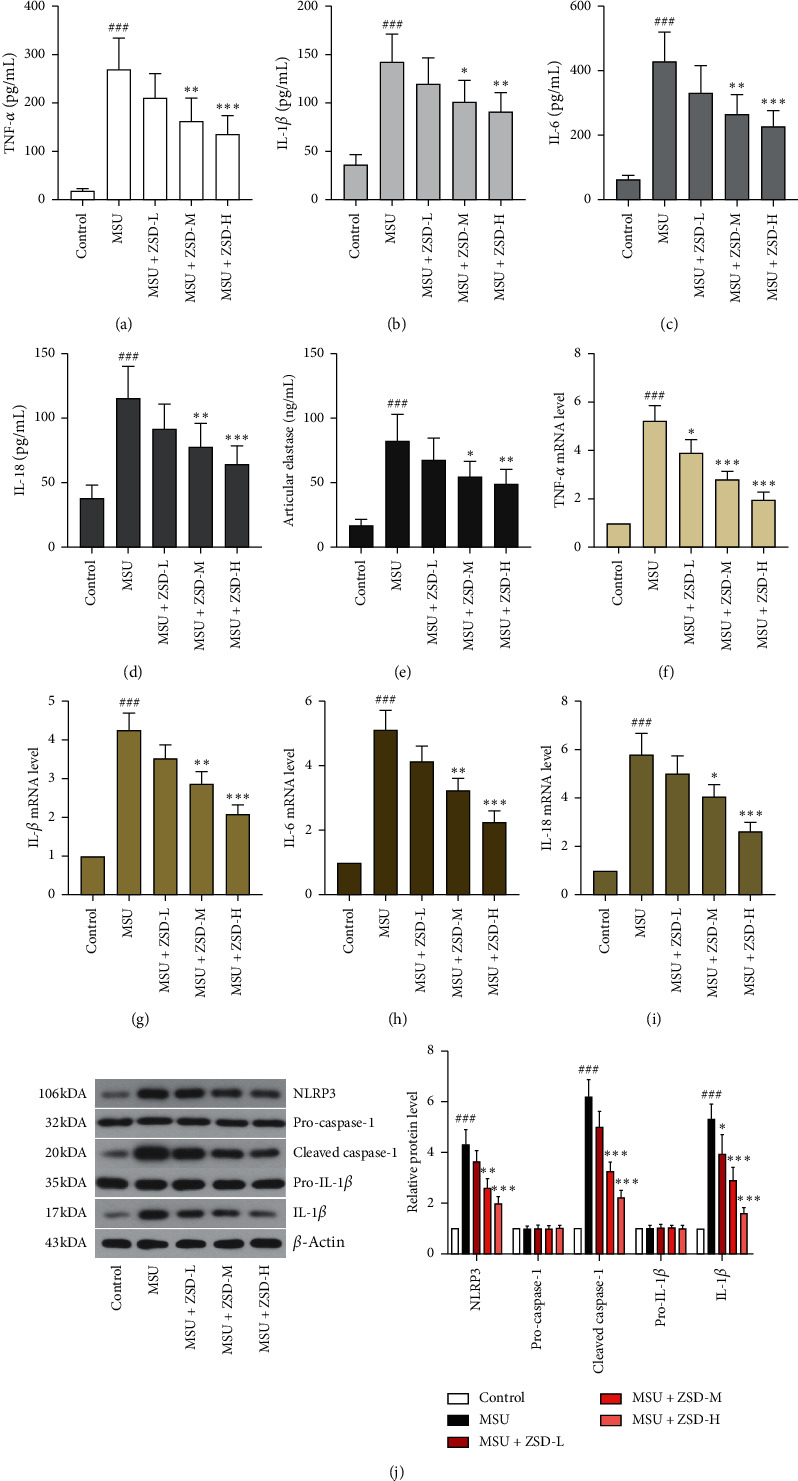
ZSD inhibited inflammation mediated by MSU in rats. ((a)–(d)) The levels of TNF-*α*, IL-1*β*, IL-6, and IL-18 in rat serum were determined via ELISA. (e) The activity of articular elastase in the rat ankle joint was evaluated with the neutrophil elastase (ELA2) detection kit. Results were presented as means ± SD. ((f)–(i)) The mRNA levels of TNF-*α*, IL-1*β*, IL-6, and IL-18 in the rat ankle joint were assessed by qRT-PCR. *β*-Actin was used as an internal reference. (j) The expressions of NLRP3, pro-caspase-1, cleaved caspase-1, pro-IL-1*β*, and IL-1*β* were detected by western blotting. *β*-Actin was used as an internal reference. Results were presented as means ± SD. ^###^*P* < 0.001 compared with the control group; ^*∗*^*P* < 0.05, ^*∗∗*^*P* < 0.01, and ^*∗∗∗*^*P* < 0.001 compared with the MSU group. TNF-*α*: tumor necrosis factor-*α*; IL-1*β*: interleukin-1*β*; ELISA: enzyme-linked immunosorbent assay; qRT-PCR: quantitative real-time polymerase chain reaction; NLRP3: NOD-like receptor protein 3.

In order to assess the role of ZSD in inflammation and articular damage, we measured the levels of inflammatory factors and the activity of articular elastase in MSU-induced rats. As depicted in Figures 2(a)–2(d), TNF-*α*, IL-1*β*, IL-6, and IL-18 levels in serum were elevated after MSU treatment for 72 h when compared to the control rats. Nevertheless, administration of ZSD dose dependently lowered the increased levels of proinflammatory cytokines. Similarly, Figure 2(e) indicates that ZSD treatment obviously reduced articular elastase activity in the ankle joint of MSU-treated rats. Furthermore, mRNA levels of proinflammatory factors in ankle joints were increased in MSU-treated rats. On the contrary, ZSD administration noticeably downregulated the mRNA levels mentioned above (Figures 2(f)–2(i)). Compared with MSU-treated rats, the protein expressions of NLRP3 and cleaved caspase-1, as well as IL-1*β*, in ankle joints were suppressed via administration of ZSD in a dose-dependent manner (Figure 2(j)). Meanwhile, there were no marked changes in pro-caspase-1 and pro-IL-1*β* levels. These findings confirmed the anti-inflammatory effect of ZSD on MSU-mediated GA in rats.

### 3.3. ZSD Reduced MSU-Mediated OS

**Figure 3:**
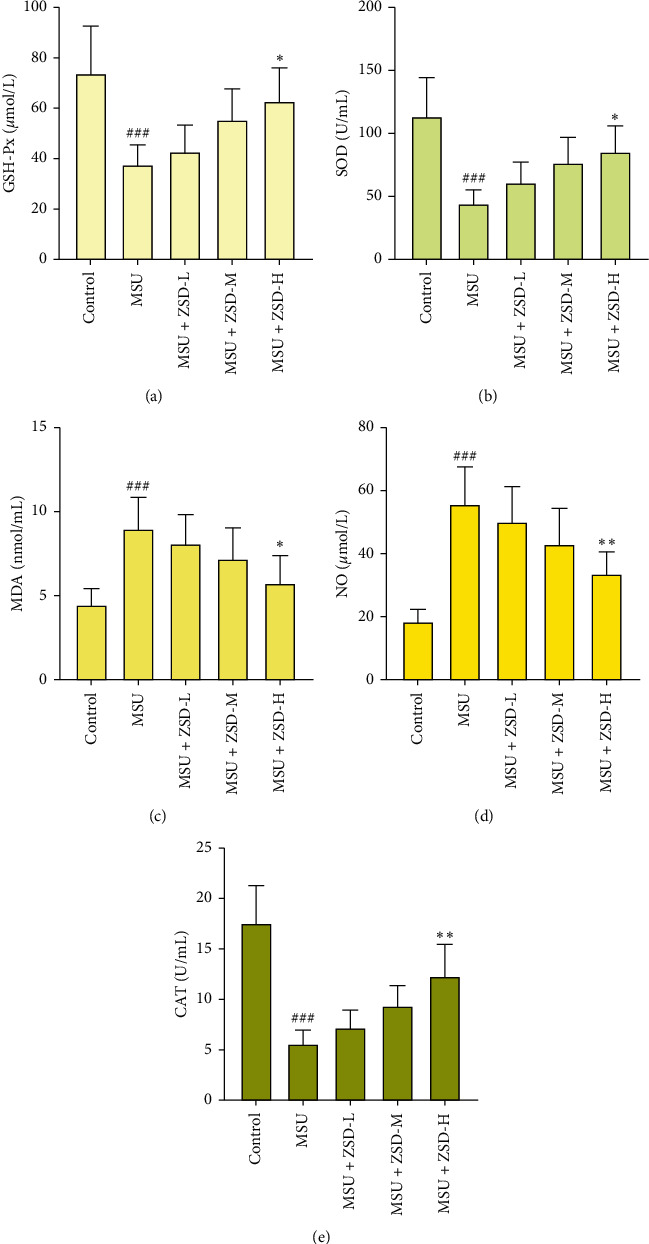
ZSD increased antioxidant status and suppressed oxidative stress in the GA rat model. ((a)–(e)) The activities of GSH-Px, SOD, and CAT, and contents of MDA and NO in serum of rats were determined using corresponding kits. Results were expressed as means ± SD. ^###^*P* < 0.001 compared with the control group; ^*∗*^*P* < 0.05, ^*∗∗*^*P* < 0.01, and ^*∗∗∗*^*P* < 0.001 compared with the MSU group. GSH-Px: glutathione peroxidase; SOD: superoxide dismutase; MDA: malondialdehyde; NO: nitrogen monoxide; CAT: catalase.

**Figure 4:**
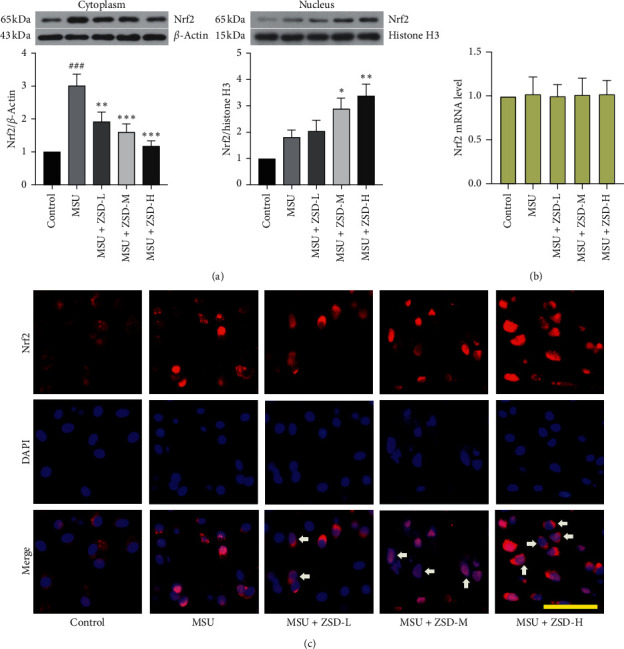
ZSD increased Nrf2 activation. (a) The expression of Nrf2 in the cytoplasm and nucleus of the rat ankle joint was evaluated utilizing western blotting, respectively. *β*-Actin and histone H3 were utilized as internal references, respectively. (b) The Nrf2 mRNA level in the rat ankle joint was detected by qRT-PCR. *β*-Actin was utilized as an internal control. Results were expressed as means ± SD. ^###^*P* < 0.001 compared with the control group; ^*∗*^*P* < 0.05, ^*∗∗*^*P* < 0.01, and ^*∗∗∗*^*P* < 0.001 compared with the MSU group. (c) The level of Nrf2 in the rat ankle joint was assessed via the immunofluorescence assay. The scale bar = 50 *μ*m. Arrows represented Nrf2-positive cells. Nrf2: nuclear factor-E2-related factor 2.

To explore the effect of ZSD on OS, some indices associated with OS were determined in GA rats. It can be seen from Figures 3(a), 3(b), and 3(e) that MSU administration for 72 h resulted in a statistically significant decrease in GSH-Px, SOD, and CAT activities; however, treatment with ZSD dose dependently increased them in serum of treated rats. At the same time, ZSD administration facilitated a significant reduction on the contents of MDA and NO in serum (Figures 3(c) and 3(d)). Moreover, as displayed in Figure 4(a), the expressions of nuclear factor-E2-related factor 2 (Nrf2) in the cytoplasm and nucleus in ankle joints were upregulated following MSU treatment when compared to the control rats. In contrast, administration of ZSD elevated the level of nuclear Nrf2, but the expression of Nrf2 in the cytoplasm was lowered.  Figure 4(b) shows that no pronounced alterations in the Nrf2 mRNA level were observed before and after treatment. Apart from these, immunofluorescence assay revealed that the nuclear translocation of Nrf2 in the ankle joint was enhanced by ZSD treatment (Figure 4(c)). These results indicated that ZSD alleviated OS and demonstrated the antioxidative property of ZSD.

### 3.4. ZSD Promoted MSU-Induced Autophagy

**Figure 5:**
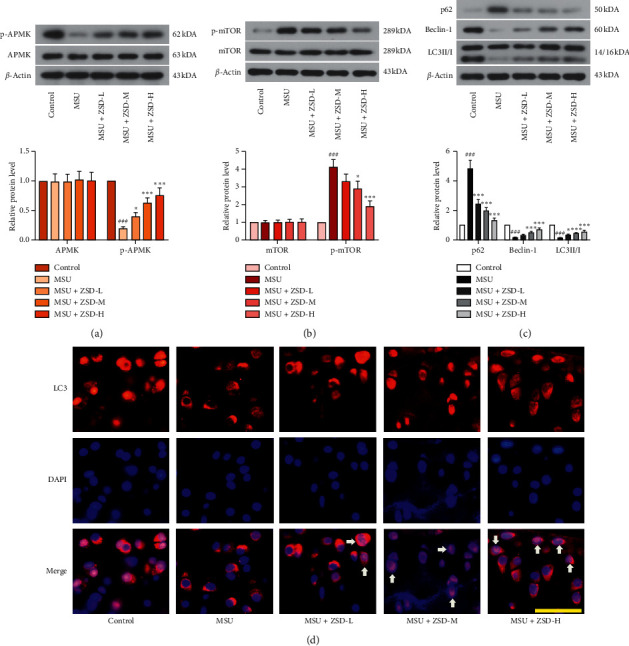
ZSD promoted tissue autophagy in MSU-treated rats. ((a)–(c)) The levels of p-AMPK, AMPK, p-mTOR, mTOR, p62, Beclin-1, and LC3II/I in the rat ankle joint were detected by western blotting. *β*-Actin was used as an internal control. Results were presented as means ± SD. ^###^*P* < 0.001 compared with the control group; ^*∗*^*P* < 0.05 and ^*∗∗∗*^*P* < 0.001 compared with the MSU group. (d) The level of LC3 in the rat ankle joint was evaluated by the immunofluorescence assay. The scale bar = 50 *μ*m. Arrows represented LC3-positive cells. AMPK: AMP-activated protein kinase; p-: phosphorylated; mTOR: mammalian target of rapamyclin.

This study was performed to confirm whether ZSD could promote autophagy. It was found that MSU treatment decreased the protein expressions of p-AMPK, Beclin-1, and LC3II/I, but increased p-mTOR and p62 levels in ankle joints of rats (Figures 5(a)–5(c)). However, administration of ZSD reversed these changes in MSU-treated rats. At the same time, there were no significant alterations in the levels of AMPK and mTOR. In addition, immunofluorescence assay was carried out to detect the localization of LC3 in the ankle joint. As depicted in Figure 5(d), the control or MSU group showed little staining of LC3, whereas ZSD administration reflected an obvious staining distribution of LC3. These findings suggested that ZSD activated the AMPK signaling pathway and repressed the activation of the mTOR pathway, thus facilitating autophagy in MSU-induced GA.

## 4. Discussion

In this study, our pathological results showed that ZSD markedly reduced the thickness and swelling degree of the right paw and inflammatory cell infiltration. The anti-inflammatory and antioxidative properties of ZSD on MSU-mediated GA in rats were validated with reference to proinflammatory factors, articular elastase, and some indicators related to OS. Noticeably, the role of ZSD in MSU-induced autophagy via the AMPK/mTOR signaling pathway was investigated.

GA is mediated by the deposition of MSU crystals in the joints of patients with hyperuricemia [14]. Our study found that MSU induced rat paw thickness and reached a peak at 24 h and then gradually decreased, which is in accordance with the study of Dhanasekar et al. [13, 15]. Besides, MSU resulted in enlarged joint space, inflammatory cell infiltration, and cartilage erosion. The above results indicate the successful establishment of the GA rat model. Moreover, It is generally agreed that MSU induces the generation of some proinflammatory mediators (such as TNF-*α*, IL-1*β*, IL-6, and IL-18) and can activate proinflammatory pathways in chondrocytes and synoviocytes [16]. We demonstrated that GA increases these levels in serum of rats, which was in line with the above finding. In addition, the interplay of MSU and macrophages in the joints results in NLRP3 activation, which triggers the cleavage of pro-caspase-1 to caspase-1 [17]. Caspase-1 cleaves pro-IL-1*β* to generate active IL-1*β*, which is the most important inflammatory downstream mediator in GA [18]. Also, neutrophil elastase, a granule serine protease, hydrolytically degrades connective tissue components including elastin and proteoglycans, as well as collagen [17]. Infiltration of neutrophil has been reported to cut precursor IL-1*β*, facilitating more neutrophils to invade and aggravate the joint, which released excessive elastase to lead to tissue damage [19]. Dhanasekar and Rasool [13] demonstrated that morin, a bioflavonol, inhibited MSU crystal-induced inflammation in an animal model of GA with regard to NLRP3 inflammasome and inflammatory factors. In the current study, our findings that ZSD decreased articular elastase activity and the protein levels of NLRP3 and cleaved caspase-1, as well as IL-1*β*, demonstrated that ZSD possessed anti-inflammatory property, which was similar to the previous report [12].

It has been accepted that the overproduction of ROS in inflammation can cause OS, leading to joint injury [13]. OS is referred to be the imbalance in the oxidative and antioxidative systems, which is able to mediate autophagy [20]. Antioxidant enzymes such as catalase (CAT), SOD, and GSH-Px fight against OS and lipid peroxidation via eliminating free radicals [8]. Additionally, ROS degrade polyunsaturated lipids to form MDA, a biomarker of oxidative damage [14]. Excess NO has been reported to be generated through elevated inducible nitric oxide synthase level in monocytes and macrophages triggered by MSU crystals [17]. Furthermore, Nrf2-mediated antioxidant pathway is tightly correlated with OS [21]. ROS generation inside the cells can be controlled via the activation of Nrf2, an important transcription factor. It is well known that Nrf2 modulates the redox status through upregulating SOD, GSH-Px, CAT, and heme oxygenase-1 (HO-1) levels [7]. In our study, ZSD increased SOD, GSH-Px, and CAT activities but reduced MDA and NO contents, which was in agreement with the previous research [12]. Nevertheless, the result that the expression of Nrf2 in the nuclear was increased by ZSD in MSU-treated rats has not been reported. The above results elucidated that ZSD had antioxidative effect, possibly via activating the Nrf2 signaling pathway and enhancing the antioxidant status.

Autophagy is a critical physiologic process to maintain homeostasis by means of degrading cytosolic macromolecules, membranes, and damaged organelles, which was modulated via autophagy-related genes [22, 23]. Beclin-1 is combined with type III phosphatidylinositol 3-kinase and Vps34 to form a complex, which allows the autophagic vesicle nucleation [22]. LC3 exists in 2 forms including LC3I as well as LC3II. In the period of autophagy formation, LC3-I in the cytoplasm is changed to LC3-II located at the autophagosomes by enzymatic hydrolysis. The degree of autophagy is dependent on the ratio of LC3II/I [24]. Furthermore, p62 has been considered an essential autophagy regulator, participating in the clearance of impaired mitochondria [25]. Autophagy has been reported to modulate inflammatory response through affecting the activation of NLRP3 inflammasome and the release of IL-1*β* [26] and is one of the cellular defenses against elevated OS as well as other diverse cell stress conditions [27]. For instance, the previous research indicated that autophagy was suppressed in osteoarthritis [22], and lowered level of autophagy was associated with NLRP3 inflammasome activation and inhibition of the Nrf2 signaling pathway [23, 26, 28]. As reported by Yang et al. [29], MSU-induced IL-1*β* generation could be diminished as a consequence of the activation of autophagy. In this study, ZSD decreased NLRP3 and IL-1*β* levels and increased the level of Nrf2. Meanwhile, the expressions of Beclin-1 and LC3II/I were upregulated, whereas p62 level was downregulated by ZSD. Accordingly, we speculated that ZSD inhibited inflammation and OS probably through promotion of autophagy. Moreover, the underlying molecular mechanisms that ZSD relieved GA via facilitating autophagy were also evaluated. AMPK/mTOR is the important modulator of cellular autophagy. Hepatic protein kinase B1 is able to activate AMPK in some circumstances such as starvation and energy consumption, which negatively regulates mTOR [30]. It has been reported that lowered expression of p-mTOR induced by AMPK phosphorylation is capable of facilitating autophagy in a variety of cell types [31]. Terkeltaub [28] reported that p-AMPK significantly suppressed mononuclear phagocyte responses to urate crystals *in vitro*, containing NLRP3 inflammasome activation and the release of IL-1*β* and chemokines. The previous work showed that hydrogen-rich saline protected lipopolysaccharide-induced acute lung injury via controlling autophagy through repression of mTOR signaling pathway activation [32]. Our experiment results suggested that ZSD activated the AMPK pathway and inhibited the mTOR pathway to facilitate autophagy. As we all know, this finding that ZSD promotes autophagy in MSU-treated rats is the first found.

## 5. Conclusions

In summary, this study implied that ZSD ameliorated MSU-induced GA in rats. The mechanism of action of ZSD was probably through inhibition of inflammation and OS and promotion of autophagy via regulation of the AMPK/mTOR signaling pathway. Therefore, ZSD, due to its anti-inflammatory and antioxidative functions, could be acted as a potential drug for preventing GA.

## Figures and Tables

**Table 1:** Primer sequences used for qRT-PCR.

Gene	Forward primer	Reverse primer
*β*-Actin	5′-GGAGATTACTGCCCTGGCTCCTAGC-3′	5′-GGCCGGACTCATCGTACTCCTGCTT-3′
TNF-*α*	5′-GCCACCACGCTCTTCTGTC-3′	5′-GCTACGGGCTTGTCACTCG-3′
IL-1*β*	5′-GGGATGATGACGACCTGC-3′	5′-ACTTGTTGGCTTATGTTCTG-3′
IL-6	5′-AACTCCATCTGCCCTTCA-3′	5′-CTGTTGTGGGTGGTATCCTC-3′
IL-18	5′-GCAGTAATACGGAGCATAAA-3′	5′-ATCCTTCACAGATAGGGTCA-3′
Nrf2	5′-TCTGACTCCGGCATTTCACT-3′	5′-TGTTGGCTGTGCTTTAGGTC-3

TNF-*α*: tumor necrosis factor-*α*; IL-1*β*: interleukin-1*β*; IL-6: interleukin-6; IL-8: interleukin-8; Nrf2: nuclear factor-E2-related factor 2.

**Table 2:** The information of antibodies used for western blotting.

Antibody	Catalog number	Dilution	Manufacturer
NLRP3	WL02635	1 : 500	Wanleibio
Pro-caspase-1	WL02117	1 : 1000	Wanleibio
Cleaved caspase-1	WL02117	1 : 1000	Wanleibio
Pro-IL-1*β*	WL02257	1 : 500	Wanleibio
IL-1*β*	WL00891	1 : 500	Wanleibio
Nrf2	WL02135	1 : 500	Wanleibio
AMPK	WL02254	1 : 500	Wanleibio
p-AMPK	2531S	1 : 1000	CST
mTOR	WL02477	1 : 500	Wanleibio
p-mTOR	WL03694	1 : 500	Wanleibio
p62	WL02385	1 : 1000	Wanleibio
Beclin-1	WL02508	1 : 500	Wanleibio
LC3II/I	WL01506	1 : 400	Wanleibio
*β*-Actin	WL01845	1 : 1000	Wanleibio
Histone H3	WL0984a	1 : 500	Wanleibio
Goat anti-rabbit IgG-HRP	WLA023	1 : 5000	Wanleibio

NLRP3: NOD-like receptor protein 3; pro-IL-1*β*: pro-interleukin-1*β*; AMPK: AMP-activated protein kinase; p-: phosphorylated; mTOR: mammalian target of rapamyclin.

## Data Availability

The data used to support the findings of this study are included within the article.
